# Sensor Network Architectures for Monitoring Underwater Pipelines

**DOI:** 10.3390/s111110738

**Published:** 2011-11-15

**Authors:** Nader Mohamed, Imad Jawhar, Jameela Al-Jaroodi, Liren Zhang

**Affiliations:** Faculty of Information Technology, P.O. Box 17551, UAEU, Al Ain, UAE; E-Mails: ijawhar@uaeu.ac.ae (I.J.); jaljaroodi@gmail.com (J.A.-J.); lzhang@uaeu.ac.ae (L.Z.)

**Keywords:** underwater pipelines monitoring, wireless sensor networks, underwater acoustic wireless sensor networks, reliable network architectures

## Abstract

This paper develops and compares different sensor network architecture designs that can be used for monitoring underwater pipeline infrastructures. These architectures are underwater wired sensor networks, underwater acoustic wireless sensor networks, RF (Radio Frequency) wireless sensor networks, integrated wired/acoustic wireless sensor networks, and integrated wired/RF wireless sensor networks. The paper also discusses the reliability challenges and enhancement approaches for these network architectures. The reliability evaluation, characteristics, advantages, and disadvantages among these architectures are discussed and compared. Three reliability factors are used for the discussion and comparison: the network connectivity, the continuity of power supply for the network, and the physical network security. In addition, the paper also develops and evaluates a hierarchical sensor network framework for underwater pipeline monitoring.

## 1. Introduction

With the increasing demand for energy and water in the World; petroleum, natural gas, and water resources and facilities have become important assets for most countries. Maintaining the economic progress of most countries is strongly dependant on maintaining and protecting these resources and facilities. One of the main and important facilities for these resources are the pipelines used to transfer water, petroleum, and natural gas. These pipelines are considered one of the main infrastructures between producer and consumer countries. Protecting the pipeline infrastructures is one of the main issues facing these countries. Furthermore, oil and gas industries in the World heavily depend on pipelines for connecting shipping ports, refineries, oil and gas wells, and power plants. For example, there are around 500,000 miles of oil and gas pipelines in the United States that also extend into Canada and Mexico [1]. These pipelines play a critical role in the U.S. economy. This pipeline infrastructure is mainly for providing energy supply to the U.S. [2].

Pipelines can be installed above the ground, under the ground, or underwater. Several long underwater pipeline systems are used for different applications around the World. One of the longest pipelines in use is the Langeled Pipeline that extends for 1,200 km from the Ormen Lange field in Norway to the Easington Gas Terminal in England under the North Sea and used to transfer natural gas to England [3]. This pipeline started operating in October 2007 and can carry 25.5 billion cubic meters per year and supplies around 20% of the natural gas demand in England. Another long pipeline is located between Qatar and UAE under the Arabian Gulf and owned by Dolphin Energy Limited of Abu Dhabi [4]. It is used to transfer processed gas from Qatar’s offshore North field to the UAE. It extends for 364 km through the Gulf and transfers a high percentage of UAE’s gas needs. In addition, pipelines are intensively used in the Gulf of Mexico to transfer oil. There are around 30,000 miles of underwater pipelines in the Gulf of Mexico [5].

Most existing and planned underwater pipeline projects are considered important infrastructures for economic stability and growth. Having a reliable monitoring and control system for these infrastructures can significantly help in inspecting and saving them. One of main approaches used to monitor different types of pipelines is sensor networks. This paper develops different sensor network architecture designs for monitoring underwater pipeline infrastructures. The developed architectures are underwater wired sensor networks, underwater acoustic wireless sensor networks, RF wireless sensor networks, integrated wired/acoustic wireless sensor networks, and integrated wired/RF wireless sensor networks. The paper compares and discusses the reliability of these proposed architectures. Although the main reason of having reliable sensor networks for monitoring is to protect underwater pipelines, the main focus of this paper is on the reliability of the sensor networks used for underwater monitoring and not on the physical pipeline protection. Three reliability factors are used to compare the architectures in terms of network connectivity, continuity of power supply for the network, and the physical network security. The paper develops an analytical model to evaluate and compare the network connectivity of the developed architectures. In addition, the advantages and disadvantages of the architectures are discussed. The paper also develops and evaluates a hierarchical sensor network framework for underwater pipeline monitoring.

The rest of the paper is organized as follows. Section 2 discusses the related work. Section 3 discusses different sensor network architectures for monitoring underwater pipelines. Section 4 develops an analytical model that evaluates the reliability of these architectures. Sections 5 and 6 develop and evaluate a hierarchical sensor network design for monitoring underwater pipelines. Section 7 provides a discussion and comparison of these architectures. Section 8 concludes the paper and highlights some of our planned future work.

## 2. Background and Related Work

Two types of threats may occur in pipeline infrastructures: intentional and non-intentional. Intentional threats can be for reasons like terrorism or illegal tapping. Pipelines in the Middle East for example are principally at risk of terrorist attacks. This is one of the main ongoing security problems in Iraq. In another example, in 2002 there were over 900 attacks on the Cano Limon oil pipeline that caused losses of around 2.5 million barrels of crude oil [6]. In addition, the pipeline was out of service for 266 days due to the fact that part of the pipeline were blown up some 170 times in 2001. The Cano Limon oil pipeline is owned by Occidental Petroleum Corp and the Colombian state oil company Ecopetrol. It transports around 110,000 barrels of crude oil a day from the Cano Limon field to the Caribbean coastal town of Covenas. Oil pipelines have also been repeatedly attacked in Nigeria. In some cases the attacks caused major damages and death of some people. The problem of illegal tapping is known in the South East Asia region. In one case a company was losing about $4m worth of oil a year through illegal tapping from an underwater pipeline [6].

Non-intentional threats may occur due to accidents such ships crashing into a pipeline, human mistakes in the pipeline operation or maintenance, or natural disasters such as volcanoes and earthquakes. For example, several underwater pipelines in the Gulf of Mexico were damaged by hurricanes in 2005 [5]. It was a very difficult and time consuming process to inspect the pipelines and find the locations and types of damages inflected by the hurricanes. Non-intentional threats can also happen due to defects in the pipeline systems. These defects can be leakage or high pressure in the pipelines. Any defect or damage in underwater pipelines may result in major environmental and economic consequences. To reduce the impact of these consequences, underwater monitoring systems can be used. These systems can provide effective and fast detection mechanisms to discover defects and respond to them in a timely and more effective manner.

There are a number of technologies to monitor, maintain and protect pipelines. Examples of these technologies are sensors, mobile robots, algorithms [7–10]. Most of these technologies are designed specifically for detecting and locating pipeline leakage [11,12], corrosion, and damage. These technologies were designed to provide a remote facility to detect and to report the positions of any defect. Some of these available solutions rely on the availability of a network to transfer the information and report the defections or any important sensed information [13]. These networks are usually wired using copper or fiber optic cables [14,15]. These wired networks are usually connected to regular sensor devices that measure specific attributes such as flow rate, pressure, temperature, *etc*.

There were some efforts to develop algorithms and methods for detecting defects such as leakages in pipelines. These algorithms and methods are based on the availability of networks along the pipelines. All these efforts are not to develop reliable and fault tolerant networks that monitor pipelines as we discussed in this paper, but rely on the existence of reliable networks. One example is PipeNet [16]. PipeNet is a wireless sensor network for monitoring large diameter bulk-water transmission pipelines. The network collects hydraulic and acoustic/vibration data at high-sampling rates. Algorithms for analyzing the collected data to detect and locate leaks were developed. In [17], a method was developed to detect faults for oil pipelines. In this method, Rough Set was used to reduce the parameters of a pipeline system. Artificial Neural Network (ANN) with three levels is used to form a detection model. In addition, a general framework using acoustic sensor networks to provide continuous monitoring and inspection of pipeline defects was developed [18]. In this framework sensor networks can detect, localize, and quantify bursts, leaks, and other anomalies in pipelines. Acoustic wave propagation theory, distributed control, and statistical signal processing are used to analyze signals for defects detection and localization. All these methods can be also used with the sensor network architectures proposed in this paper.

In another project, a wireless sensor network for a team of underwater collaborative autonomous agents has been developed [19]. This system was developed to locate and repair scale formations in tanks and pipeline within inaccessible areas such as underwater environments. We have previously developed a framework and protocols for monitoring above-ground long pipelines using wireless sensor networks [20]. In addition, we have developed a fault-tolerant wired/wireless sensor network architecture for monitoring above-ground pipeline infrastructures [21]. Although several of the mentioned projects are based on different network technologies, none of them studied the reliability issues of sensor networks for monitoring long underwater pipelines.

Some companies have developed sensors for monitoring pipelines. Most of these commercial sensors are developed to provide individual sensing for some attributes. An example of these companies is vMonitor [22], which developed a set of wireless pressure and temperature sensors to remotely monitor pipelines. These sensors were designed mainly for above ground pipelines. Another example is from Westminster International LTD, which developed Diver Detection Sonar (DDS) that can be used underwater for the detection of divers and submerged swimmer delivery vehicles (SDVs) [23]. These sensors can be used to counter the threat of sabotage and terrorism for naval bases, commercial ports, as well as oil platforms by detecting unwanted divers, swimmer, and SDV. These commercial products can also be used as part of some of network architectures developed in this paper for monitoring underwater pipelines.

## 3. Sensor Network Architectures for Monitoring Underwater Pipelines

This section develops a number of sensor network architectures that can be used to monitor underwater pipelines. In this section we discuss these architectures and show their advantages, disadvantages, and challenges. These networks can be used to allow a remote facility to detect and to report the positions of any leakage, defect, or risk. One of the main differences between the networks used for pipelines and other networks is that the network needed for pipeline applications is structured in a line where all sensor nodes are distributed on that line. This characteristic enforces some reliability challenges in monitoring pipeline infrastructures. The reliability challenges are linked directly with the reliability of the network connecting these sensor nodes. Having a reliable network is one of the main conditions of having a reliable monitoring system for pipelines.

Different network architectures are developed in this paper for reliable communication in pipeline systems. These architectures are based on wired networks, wireless networks, or a combination of wired and wireless networks. These architectures are evaluated based on three reliability factors:

1. *The Connectivity of the Network*: since the pipeline network extends linearly, it is important for the network to be continuously connected to transfer information from the sensor nodes distributed across the pipeline to the Network Control Center (NCC), that provides the main control for the underwater pipeline network, and also to transfer control commands from the NCC or other control points to the actor and sensor nodes.
2. *The Continuity of Power Supply*: pipeline networks will not be able to operate unless there is a sufficient power supply available continuously. Power is needed not only to operate the network but also to operate the sensor and actor nodes.
3. *The Physical Network Security*: pipelines are usually considered important infrastructures. Devices or networks monitoring these infrastructures must be physically secured. Otherwise, the monitoring systems will fail easily and the pipelines will not be appropriately protected.

These important reliability factors are chosen in this paper based on the characteristics of the targeted environment. First, existing and planned underwater pipelines expand for tens and hundreds of kilometers in unattended and technically challenging environments. In this type of environments, it is not easy to find and fix network faults. Fixing network faults underwater may take a very long time during which the pipelines will be fully or partially unmonitored. Networks in this type of environments are structured linearly where all sensor and actor nodes are distributed along a somewhat single line. These devices will not be able to communicate with the NCC unless the network connectivity along the line is available. Different sensor network architectures can provide different levels of connectivity in the normal case and with the existence of network faults. Generally, it is important to maintain some level of connectivity even with occurring network faults. Second, sensor, control, and other network related devices on the used need power to operate and the network expands on very long distances thus maintaining the availability of power to operate the network is an important factor for maintaining its reliability. Some network architectures can provide continuous power supply for its components while others rely on batteries, which limit the life of the network. Third, as this type of networks is in unattended environments, the reliability of the networks depends on the physical protection of the network components. Different sensor network architectures have different degrees of physical protection, thus, different levels of reliability. Some network architectures can be completely hidden while others have some components that are physically visible, which makes them susceptible to attacks or accidents.

### 3.1. Underwater Wired Sensor Networks

**Figure 1.**
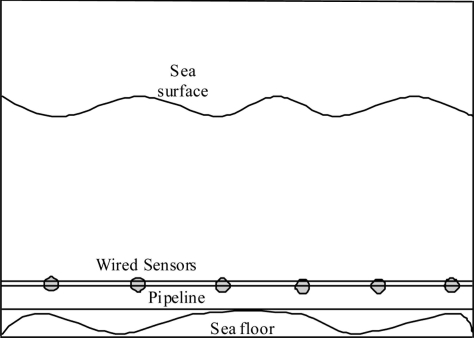
Underwater wired sensor network.

Currently, most pipeline sensors are connected using wired networks. Wired networks are either copper or fiber optic cables. The wired networks are usually connected to regular sensor devices that measure specific attributes such as flow rate, pressure, temperature, sound, vibration, motion, and other important attributes, see Figure 1. The wires are not used for communication only but also to transfer electrical power to different parts of the pipeline system to enable the sensors, actors, and communication devices to function. Power for the pipeline resources and networks can be provided by different sources:

1. *Solar Energy*: arrays of solar cells can be used to generate electric power for the pipeline infrastructure. This power is supplied to the different communication and sensor devices. While this option is suitable for above-ground pipelines, it is not applicable for underwater pipelines.
2. *Pipeline Flow Energy*: electric power can be generated using turbines embedded through the pipeline. These turbines rotate under the pressure of the fluid moving through the pipelines and generate electric power. This can be used for the different sensor and communication devices installed along the pipeline [24].
3. *Other External Energy*: power can be provided from external sources such as external gas-based power generators or third-party power generators. This power is transferred in wires to different communication and sensor devices along the pipeline.

Wired networks are considered the traditional way for communication in pipeline systems. They are easy to install and provide power supply for through the network wires. However, there are a number of reliability problems related to using wired networks with regular sensors for monitoring pipelines. These problems are:

▪ If there is any damage in any part of the wires of the network, the pipeline communication system will be completely or partially damaged. This depends on how the wired network is organized and used. If the communication is done in one direction on the wire, then a single cut on the wire will disconnect all the nodes after the cut from the NCC. If the communication is two-directional then the negative impact on the communication is less as some nodes will use one direction for communication while the nodes after the cut can use the other direction. In this case the NCC needs to be connected to both ends of the network. However, if there are two or more cuts in the network, then all nodes between the cuts will not be able to communicate with either of the NCC. In addition, if there is a power outage, some of the nodes may not be able to operate.
▪ It is easy for unauthorized people to disable the network simply by cutting some of the network wires.

These problems make wired networks for pipeline communication systems undependable. Although, wired networks provide an easy solution for underwater pipeline monitoring and controlling, they face a number of major reliability and security problems. The main reason of these problems is the structure and type of networks used for pipelines. If any part of the wired network is disabled for any intentional or natural reason, the monitoring system can be partially or completely affected.

One possible solution to enhance the reliability of a wired network is to use multiple networks that expand through the whole area. One of these networks will be used as primary while others are kept as backup. However, unlike other systems, using multiple wired networks and a fault tolerance mechanism among them will not enhance the reliability of the pipelines network. This is due to the fact that all wires will be expanded together along the pipelines and any damage occurring in one of them may also occur in the others. More specifically an accidental (or intentional) break in the line has a very high probability of happening to all wires along the pipelines.

### 3.2. Underwater Acoustic Wireless Sensor Networks

Acoustic wireless sensor nodes can be installed with underwater pipeline infrastructures. Each node has a limited transmission capability in which each node can communicate with few neighboring nodes. Multi-hop communication is used to transfer the sensed information among the underwater pipeline.

**Figure 2.**
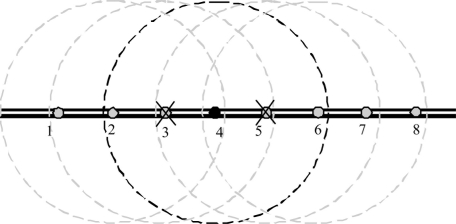
Reliability in dense sensor networks.

Wireless networks can solve some of the reliability problems of current wired networks technologies in pipeline systems [20]. For example, wireless sensor networks can still function even when some nodes are disabled. Faults in sensor nodes can be easily tolerated by using other available nodes to cover the faulty ones. Using dense sensor networks with a high number of nodes and/or using wide acoustic transmission range, the network can maintain connectivity and the sensed information can be transported through the network to its destination even with the existence of some node failures. For example, each node in Figure 2 can communicate with two nodes to the left and two nodes to the right. If for example node 3 and 5 are damaged, node 4 can still send its sensed data through nodes 2 or 6.

Each sensor node for monitoring pipelines is usually equipped with an acoustic transceiver, a processor, a battery, memory, and small storage in addition to one or more sensor devices. Power consumption is critical to the life span of pipeline communication systems. Pipeline systems are usually installed to be used for many years. Therefore the associated communication systems should also be long lived. Unlike wired networks where the power is not at all a constraint in building the system, network designers have to consider power as one of the main constraints in the wireless system. Power in a node can be consumed when data is sent through the transceiver, when the transceiver is turned on waiting to receive data from other nodes, when sensor devices are turned on, and when the processor is active. Careful scheduling of these resources is needed to optimize power consumption.

**Figure 3.**
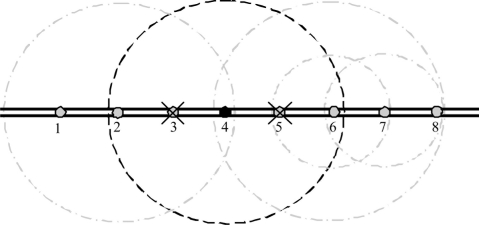
Automatic wireless/acoustic range.

Although increasing the range can provide better reliability, more energy will be consumed from the nodes. A dynamic configuration for the wireless transmission range can provide better power management. Example of this configuration is in Figure 3. In this network, nodes 3 and 5 are dead. Therefore, the wireless range for node 4 is increased to reach nodes 2 and 6 while other nodes use a smaller transmission range to reduce the power consumption.

In addition, nodes are used to route information from other nodes to the NCC. As a result, nodes close to the NCC will consume more power than other nodes since they will route more packets. All nodes will have the same level of sensing activity; however, closer nodes to the NCC will consume more power due to more packet routings. One of the main issues for wireless/acoustic networks when used to monitor pipelines is the optimal design of a network protocol that balances the power consumption of batteries on the nodes with and without node failures. This balancing is crucial to extend the life of the network.

**Figure 4.**
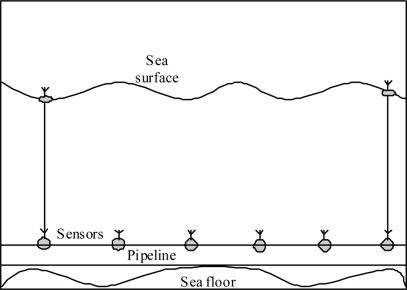
Underwater acoustic sensor network.

One approach to enhance the reliability of underwater acoustic sensor networks for pipelines is to divide the network into multiple parts. In each part, there is a surface buoy that is linked with one of the underwater sensor nodes through a wire as shown in Figure 4. Each surface buoy is equipped with a radio communication system to transfer collected information to the NCC. The collected information can be transferred either directly through satellite communication links or using radio multi-hop communication among the available surface buoys. All acoustic sensor nodes need to transfer their sensed information to the nearest neighboring node linked to one of the surface buoys.

Generally, underwater acoustic networks suffer some major problems [25–27]. First, the available bandwidth is very limited among the nodes. The bandwidth of underwater acoustic channels depends on both the distance and frequency [28]. Communication among nodes located within the range of a few tens of kilometers may have a bandwidth of only a few kHz. The bandwidth can be increased to a hundred kHz, if the nodes involved are located closer (*i.e*., within several tens of meters). Second, propagation delay of acoustic signals underwater is very high and variable [29]. It is around five orders of magnitude higher than that in radio frequency channels. Third, due to the characteristics of the underwater channel, high bit error rates and connectivity losses can be encountered. Forth, battery power is consumed and it is difficult to renew or replace. In addition to the power needed to operate the nodes and their transceivers, in underwater communication advanced signal processing techniques are needed to reduce the impact of underwater communication characteristics; however, these signal processing techniques will consume more power and will reduce the life of the nodes’ batteries.

Acoustic communication is used for monitoring vibrations in the Langeled Pipeline installed at a depth of 800–1,100 meters on a hilly and rocky seabed. Several segments of the pipeline are not in contact with the seabed. With strong sea currents, high vibrations may be induced in these free spans. This introduces high and risky pressure on the pipeline segments. Consequently, it was a requirement to monitor vibrations on the pipeline. A wireless sensor network was installed on the pipeline to monitor vibrations [30]. The monitoring network consists of autonomous synchronized wireless acoustic nodes. These nodes use acoustic Clamp Sensor Packages (CSP) that are mounted to the pipeline at regular intervals and a Master Sensor Packages (MSP) that monitors the vibrations in longer pipeline free spans. The CPSs are equipped with batteries that last for six months. Remote operating vehicles (ROV) are used to replace dead nodes. This type of monitoring network requires periodic and costly maintenance due to the use of batteries for operating the nodes.

### 3.3. RF Wireless Sensor Networks

**Figure 5.**
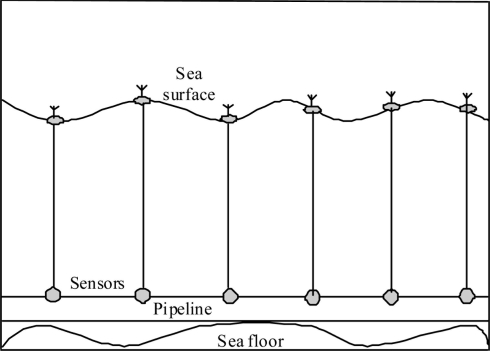
RF Wireless sensor network.

Another architecture that can be used for monitoring underwater pipelines is wireless sensor networks that use radio frequency. The main differences between this architecture and other wireless sensor networks used for above ground applications is that each sensor node is connected to a surface buoy through a cable as shown in Figure 5. Radio transceivers are available on the surface buoys. As a result, the nodes can communicate through radio frequency channels which provide better communication bandwidth, propagation delay, bit error rate and connectivity, as well as less power consumption for processing communication signals compared to the underwater acoustic sensor network. In addition, surface buoys can be equipped with solar cells to provide energy for the sensors and communication devices.

Although this architecture has many advantages over the acoustic sensor network, it has two major physical security problems. First, the floating buoys and their cables may be damaged by passing ships. In addition, the locations of the floating buoys are a good indication for the location of the pipeline. Therefore, the pipeline can be easily discovered and sabotaged. In addition, using buoys connected through wires to an underwater pipeline in very deep seas is not a practically feasible solution. These wires will be vertically expanded and as the length increases to the sea surface, the probability that the wires will be damaged (accidentally or due to sea currents) also increases.

### 3.4. Integrated Wired/Acoustic Wireless Sensor Networks

**Figure 6.**
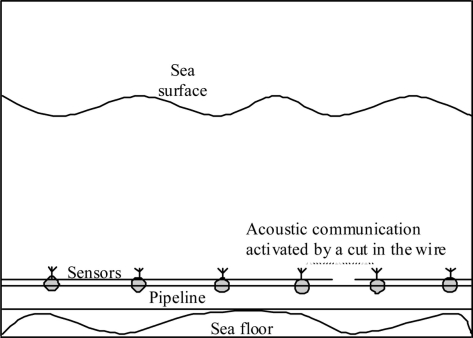
Integrated wired/acoustic wireless sensor network.

As we can see from the previous sections the main reliability challenges in pipeline sensor networks communication systems are network connectivity, continuity of power supply, and physical security of the network. To solve all these issues, we are proposing in this section new network architecture for underwater pipeline communication systems. The architecture in this system consists of multiple point-to-point segments as shown in Figure 6. These segments link the system nodes.

Nodes are either sensor or actor nodes. Each node is connected to a acoustic transceiver and a wired network interface. Sensor nodes also consist of processor, memory and storage units. The nodes are connected through wireless acoustic and wired links. Wires are used for networking and for transferring power to the nodes. Unlike completely wireless nodes in the wireless or underwater acoustic sensor network architectures, nodes in this architecture have rechargeable batteries which are charged by the received power through the connected wires. The power can be provided for this network architecture using the techniques used for wired network architecture as discussed in Section 3.1.

Neighborhood nodes can communicate either using wired or acoustic communication. The transceivers in the normal case are turned off and the wired network is used for communication. Therefore the connectivity of the network is through the wired links in the normal case. Each node periodically checks the status of the right side of the network wire by sending echo messages to the neighboring nodes on the right. Each node also periodically checks the status of the left side network wire by receiving/replying to the echo messages received from the neighboring node on the left. A break of a wired link between two nodes can be discovered by the left node when it does not receive replies for the echo messages it just sent. The break can be discovered by the right node if there are no echo messages received from the left node. When both nodes discover the break, they will activate their transceivers and communicate through the acoustic link. This wireless acoustic link between the two nodes can provide connectivity for the pipeline network and sensed information can be still transported through the network as shown in Figure 6. The nodes that discovered the break will report it along with the location information to the NCC for immediate maintenance. If an intermediate node is disconnected from the left and from the right, the node can operate temporarily using the rechargeable battery until the wire breaks are fixed.

Link breaks due to faulty nodes can be recovered by using a wider transmission range in which each node can communicate using the wireless links with multiple nodes on the left and multiple nodes on the right as discussed in Section 3.2. Discovered faulty nodes can be also directly reported to the NCC. The network connectivity will remain even with multiple breaks on multiple segments occurring while any node faults or wire breaks will be discovered and reported for maintenance. In addition, with the availability of rechargeable batteries, the power constraint issue is terminated. Ordered ID codes can be used for identifying and positioning nodes along the pipeline.

One of the main disadvantages of this architecture is that with a single wire cut, the overall bandwidth of the network will drop to the bandwidth of the replacement acoustic communication channel. However, unlike with the wired sensor network solution, the communication bandwidth will not drop to zero. The limited available bandwidth can be used to report the position of the faults as well as other critical information until the damage is fixed.

### 3.5. Integrated Wired/RF Wireless Sensor Networks

**Figure 7.**
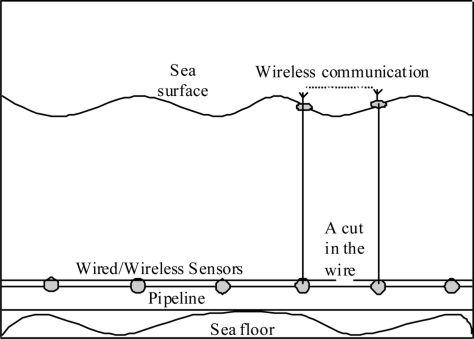
Integrated wired/RF wireless sensor network.

To enhance the communication of the architecture described in Section 3.4, radio frequency channels can be used instead of acoustic communication channels, as shown in Figure 7. In this architecture, a buoy equipped with a radio transceiver is released when there is a cut or fault in the wire connecting a pair of nodes as shown in Figure 7. The buoys are attached to the nodes and they only float on the surface if there is a need for them. The transceivers on the floating buoys are activated to provide communication and connectivity among them thus replacing the broken link underwater.

This architecture is very similar to the one discussed in the previous section; however, it can provide better communication as radio frequency channels are used instead of the acoustic channels that have lower network properties. This architecture will provide better physical security protection for the network than the RF wireless sensor network architecture described in Section 3.3 as the buoys will not appear unless there is a problem with the wired connections. In addition, only sparse pairs of buoys will be deployed at a time thus they will not reveal the full location or spread of the pipelines.

## 4. Reliability Comparison and Evaluation

Sensor networks for monitoring underwater pipelines are usually linear. Sensors can forward their sensed information either through their left side, their right side or both. In this section, we develop an analytical reliability model to analyze and compare the connectivity reliability of the different architectures discussed in this paper. Let us assume that the sensors are uniformly distributed along the pipeline. In addition, they are generating messages at the same rate. Let us assume that there are *n* sensors and *n + 1* links in the network. The sensors are *S_1_*, *S_2_*, *S_3_*,…, and *S_n_* from the left to the right of the network. In our model, we will assume that a sensor can send its sensed information to the NCC if the link from the left side of the sensor is healthy or if the link from the right side of the sensor is healthy. In other words, the availability of either connection will allow the sensor to send its information. This means that the NCC is linked to both sides of the network. We will develop a general reliability model and then we will customize that model to the different types of sensor network architectures.

To start developing the model we will consider one sensor on the network. A sensor cannot send its information to the NCC if there is no connectivity from that sensor to the NCC from the left and right sides of the sensor. If we take one side of the network, the sensed information cannot be transferred if there is a broken link which cannot be used to forward the information to next link towards the NCC. Let *L_i_* be the probability that the sensor S*_i_* cannot forward its sensed information to the NCC from the left side and *R_i_* be the probability that the sensor *S_i_* cannot forward its sensed information to the NCC from the right side. We have:

(1) $$L_{i}=\left(1-w^{i}\right)$$

(2) $$R_{i}=\left(1-w^{n-i}\right)$$

where *w* is the probability that a link or a hop can forward the information to the next link or hop. This is from the fact that a senor can send its sensed information to the NCC if and only if all links from this sensor to the NCC are operating; otherwise, when there is one or more faulty links in that direction, the sensed information will not be able to reach the NCC. Now, let *Q_i_* be the probability that sensor *S_i_* cannot send its information from both sides. We have:

(3) $$Q_{i}=L_{i}\cdot R_{i}$$

(4) $$Q_{i}=\left(1-w^{i}\right)\cdot \left(1-w^{n-i}\right)$$

Let *P_i_* be the probability that sensor *S_i_* can send its sensed information to the NCC. We have:

(5) $$P_{i}=1-Q_{i}$$

(6) $$P_{i}=1-\left(1-w^{i}\right)\cdot \left(1-w^{n-i}\right)$$

Now, the expectation for all sensors on the network to be able to send their sensed information to the NCC is:

(7) $$E_{n}=\frac{\Sigma_{i-1}^{n}P_{i}}{n}$$

(8) $$E_{n}=\frac{\Sigma_{i-1}^{n}\left(1-\left(1-w^{i}\right)\left(1-w^{n-1}\right)\right)}{n}$$

The value of *w* can be calculated in different ways in the mentioned architectures. For wired sensor networks, a long network can be divided into multiple segments. A segment has a probability *d* of being healthy. In this network, we have

(9) w=d

For the wireless sensor network:

(10) $$w=1-x^{j}$$

where *x* is the probability that a node cannot forward a message to a next hop and *j* is the maximum jump factor that a node can reach to other nodes in one direction. For example, if each sensor can jump over 3 sensors from each side, then *j = 3*. Here we have *x^j^* is the probability that a sensor cannot forward its messages in one direction; while *1 − x^j^* is the probability that a sensor can forward its messages. This is calculated from the fact that a sensor will not be able to forward its messages in one direction if and only if all next *j* sensors are faulty.

For the integrated wired/wireless sensor networks:

(11) $$w=1-\left(x^{j}\cdot \left(1-d\right)\right)$$

where *x* is the probability that a node cannot forward to a next hop, *j* is the maximum jump factor, and *d* is the probability that a wired link is healthy. We have (*1 − d*) is the probability of having a faulty wired link. A sensor will not be able to forward its messages through one direction if and only if the wired segment is broken and all next *j* sensors are faulty. Otherwise, the sensor can forward its information.

**Figure 8.**
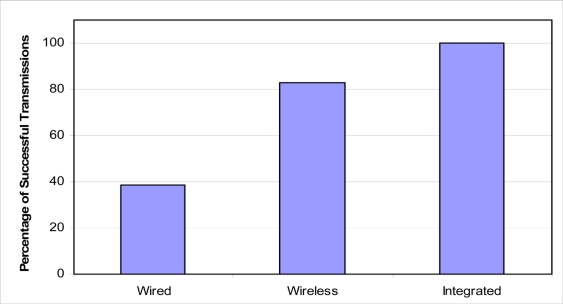
Transmission reliability for different architecture scenarios.

To evaluate the three architectures, let us assume the following scenario. We have an underwater pipeline that needs to be monitored using 500 sensors distributed uniformly. If a wired network is used to connect the sensors, let us assume that the health probability of each wired link between each neighbor pair of sensors is 0.99. If a wireless sensor network is used, let us assume that the probability that a wireless node can forward a message is 0.85, and the jump factor is 3. For the wired/wireless network, we assume having the same corresponding values as the ones used for the wired and wireless networks. Using Equation (8) where *w* is replaced by Equation (9) for the wired network, Equation (10) for the wireless network, and Equation (11) for the integrated wired/wireless network, the expectation for the network having the ability to transfer its sensed information to the NCC is shown (see Figure 8).

**Figure 9.**
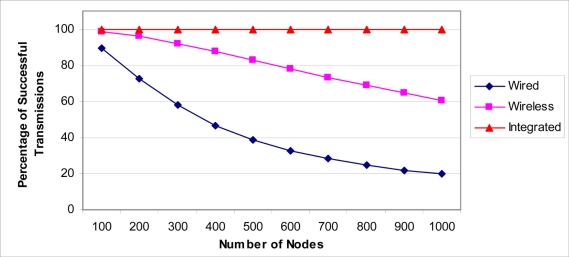
Impact of increasing the network length and the number of sensors.

On the other hand, to evaluate the impact of increasing the number of sensors on the networks and the total distance of the network, we will consider the previous example with varying numbers of sensors. The results are shown in Figure 9 for networks with 100 to 1,000 sensors. As we can see from the figure, the connectivity reliability of both wired and wireless sensor networks are significantly reduced with the increasing size of the network while the connectivity reliability was not reduced much in the integrated wired/wireless network.

## 5. Hierarchical Sensor Network Design

Although this paper provides several new architectures for underwater pipeline monitoring, it is noted that these architectures follow the linear sensor network model which we previously introduced in [31]. This general model can be adapted for the various categories of networks that were introduced. In this section, a brief description of this model is presented, and in a later section, simulation experiments performed to evaluate the performance of a specific implementation of this model will be offered. One of the important aspects of this hierarchical structure is its ability to take advantage of the linear structure to improve the reliability and robustness of the network in reaction to node failures. Even though the failures can have various causes, including intentional physical attacks, equipment failures, battery depletion (in case of battery-powered nodes), the network and corresponding message routing protocols are able to react quickly, autonomously, and efficiently to overcome the failures. In the above model, the following three types of nodes are defined. Each of these types of nodes has different functions to perform in the data collection, routing and final dissemination to the NCC.

▪ *Basic Sensor Nodes* (BSNs): These are the most common nodes in the network. Their objective is to perform the sensing function and communicate this information to the data relay nodes.
▪ *Data Relay Nodes* (DRNs): These nodes serve as information collection nodes for the data gathered by the sensor nodes in their one-hop neighborhood. They also play a role in routing the data to the NCC. The distance between these nodes is determined by the communication range of the MAC protocol used.
▪ *Data Dissemination Nodes* (DDNs): These nodes perform the function of delivering the collected data to the NCC. The technology used to transfer data from these nodes to the NCC can vary. For example, satellite cellular technology can be used. This implies that each of the DDN nodes would have such communication capability.

**Figure 10.**
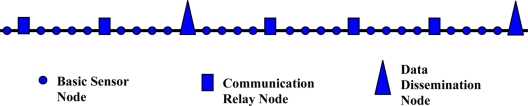
A representation of a three-level hierarchical sensor network design.

The DDNs provide the network with increased reliability since the collected sensor data would not have to travel all the way along the length of the pipeline from the sensing source to the NCC. This distance is usually very long and can be hundreds of kilometers which would make it vulnerable to a large number of possible failures, unacceptable delay, higher probability of errors, and potential security attacks. The DDNs allow the network to pass on its sensor data simultaneously in a parallel fashion. Additionally, the distance between the DDNs is important and affects the reliability of the network. A small distance between the DDNs would increase the equipment cost of the network, as well as deployment and maintenance costs. On the other hand, a distance that is too large would decrease the reliability, security, and performance of the network. Figure 10 shows a graphic representation of the different types of nodes and their geographic layout.

A hierarchical relationship exists between the various types of nodes in the sensor network. Multiple BSNs transmit their data to the nearest DRN. In turn, several DRNs transmit their data using a multi-hop strategy through the intermediate DRNs to the nearest DDN node. Finally, all DDNs transmit their data to the NCC. BSNs, DRNs, and DDNs can be completely wireless nodes that are equipped with batteries and used for underwater acoustic wireless sensor network or RF wireless sensor network architectures. BSNs, DRNs, and DDNs can be wired/wireless nodes and used for integrated wired/acoustic wireless sensor network and integrated wired/RF wireless sensor network architectures. In this case, the nodes will be equipped with rechargeable batteries that can be recharged from the wire. In addition, the wireless communication will be used only if there are some broken wires. Under normal conditions, the wire will be used for communication. In all cases, nodes can be connected to buoys on the sea surface for RF wireless communication.

## 6. Performance Evaluations

In this case study, we focus on the routing protocols that can be used to transport the collected BSN sensor data across the linear network using the DRN nodes. The presented protocols take advantage of the linear nature of the network to increase the robustness in reaction to failures, reliability, and efficiency of the routing and communication process.

Communication between the DRN and DDN nodes is done using a multi-hop routing algorithm which functions on top of a MAC protocol such as Zigbee/IEEE 802.15.4. As mentioned earlier, the DRN nodes can play the role of Zigbee FFD nodes. The Zigbee routing mechanism for the FFD nodes normally uses the AODV routing protocol where the FFD nodes act as network routers. While the AODV routing protocol is relatively effective for a multi-dimensional sensor network with an unpredictable infrastructure, it incurs too much overhead in message exchanges related to route discovery and maintenance [32–35]. In this paper, we propose two routing protocols for DRN-to-DDN communication that take advantage of the special linear structure of the network to increase routing efficiency, and reliability. When a node fails, our proposed protocols either jump over the failed node by extending the node’s transmission range, or redirect the message in the opposite direction to reach an alternative DDN node. This kind of adaptive reaction to node failures which is specific to WSNs with a linear structure is not available in AODV. Instead, in reaction to node or link failure AODV would reinitiate route discovery without extending the range of the node that is a neighbor of the failing node and would simply fail to find a new route to the target DDN node. After several failed route discoveries, which consume precious bandwidth and cause unnecessary delay, AODV will simply drop the message. AODV would not be aware of the fact that by simply extending the range of the neighboring node the target DDN node can be reached. Also, AODV would not be aware, that another alternative route to another nearby DDN node can also be used to ultimately transmit the DRN data to the NCC. These important strategies are only possible due to the special linear topology and scalable hierarchy of the network. Furthermore, as indicated in [34], when the number of nodes in the network grows, the routing table which is necessary for the proper functioning of AODV simply grows too fast in a manner that is not desirable for sensors devices which usually have a relatively small memory. The same argument can be used to show why other similar *ad hoc* and sensor network routing protocols would also not be very effective in this environment.

With some basic modifications, the hierarchical model described in the previous section can be used to represent any one of the various architectures presented earlier. For example, the underwater nodes belonging to the underwater acoustic sensor network presented in Figure 4 can be considered DRNs, and the surface buoy nodes can be considered DDNs. In addition, each of the DRNs can collect the information to be relayed along the pipeline from multiple BSNs that are local to it. On the other hand, the wireless sensor network shown in Figure 5 can be considered as consisting of DRNs that relay the information among each other until a designated node transmits the collected information to the NCC. Similar modeling can be done for the other presented architectures. In this paper, we consider the network presented in Figure 4 as a case study. Simulation experiments were performed in order to verify the operation, and evaluate the performance of the proposed. Two strategies for using multi-hop routing of data messages through the DRNs to reach the nearest DDN were compared. The two algorithms deal differently with the failure of a next-hop DRN.

### 6.1. Strategy of Jumping over Failed Nodes by Extending the Transmission Rage

In order to still be able to transmit its DRN data successfully despite the lack of connectivity to its immediate neighbor, the DRN can increase its transmission power and double its range in order to reach the DRN that follows the current one. If multiple consecutive links are lost, then the DRN can increase its transmission range appropriately in order to bypass the broken links. This process can happen until the transmission power is maximal. If even with maximal transmission power the broken links cannot be bypassed, then the message is dropped. In the protocol, this maximal DRN transmission power is represented by a network variable named MAX_JUMP_FACTOR which holds the maximum number of broken links or “disabled nodes” that a DRN transmission can bypass.

### 6.2. Redirect Always Algorithm (RA)

In this variation of the routing protocol, the DRN source node sends its DRN data message to its parent DDN. While the message is being forwarded through the intermediate DRNs, if it reaches a broken link then the following steps are taken. The DRN determines if this data message has already been redirected. This is determined by checking the redirected flag that resides in the message. If the redirected flag is already set then the message is dropped and a negative acknowledgement is sent back to the source. Otherwise, the source can be informed of the redirection process by sending a short redirection message with the redirected message ID back to the source. The source will then re-send the data message in the opposite direction and update its database with the fact that this direction to reach the DDN is not functional. Furthermore, in order to make the protocol more efficient the entire data message is not sent back to the source since the source already has a copy of the data message. Only a short redirection message with the redirected message ID is sufficient to be sent back to the source. Additionally, the redirection message also informs the other nodes on that side that there is a “dead end” in this direction and data needs to be transmitted in the other direction even if the number of hops to reach the other nearest DRN is larger. In that case, each DRN that receives this message will check the redirected flag, and if it is set, then it will continue to forward the message in the same direction. However, in order to prevent looping, if another broken link is encountered in the opposite direction the redirected message cannot be redirected again. In that case, the message is simply dropped.

### 6.3. Smart Redirect or Jump Algorithm (SRJ)

This algorithm overcomes DRN failures by using a combination of the jumping and redirection strategies described in the previous sections. We define as sibling DRNs to a particular DRN x, the DRNs that have the same parent DDN as x. We also define as secondary sibling DRNs to x the DRNs that have as parent DDN the secondary parent DDN (*i.e.*, the DDN that is on the opposite side of the parent DDN with respect to x) of x. In this algorithm, each node contains information about the operational status of its sibling and secondary sibling DRNs. Consequently, before dispatching the message, it calculates the total necessary energy it needs to reach its parent DDN Expand the total energy it needs to reach its secondary parent Exsp. It then dispatches the message in the direction which takes the lower total energy to reach either the parent DDN or the secondary parent DDN. Specifically, if Exp ≤ Exsp then the message is sent towards the parent DDN. Otherwise, the message is sent towards the secondary parent DDN. This algorithm relies on the information in the node to reduce the total energy consumed by the network for the transmission of the message. This information about failure status of DRNs is cached by the DRNs from participations in previous packet transmissions. More research is being conducted for the most efficient means of gathering such information by the DRNs.

### 6.4. Analysis of Results

Simulation experiments were performed to verify the correctness of operations, and evaluate the performance of the proposed framework and network protocol. The simulator is event-driven and it was written using Java. It places all the nodes in a linear formation, and simulates the communication and routing processes as described in the paper. The main focus of the experiments was to validate and evaluate the design and inter-workings of the proposed model. The simulator was designed and tested according to the model and structure presented in [36]. As indicated in Table 1, the number of DDN nodes used in the simulation is 10, the number of DRN nodes per DDN node was varied between 25, 50, 100, and 150. The number of BSN nodes per DRN node is 6. DRN nodes communicate using a data rate of 4,800 bps. Depending on the transmission range of the nodes, this number of nodes covers an area of tens of kilometers while the network can cover longer stretches of pipelines. However, since the hierarchical network consists of segments which are separated by the DDN nodes, the simulation of several segments of the network is relatively sufficient to reflect the performance of the entire system. This is because the data is only multi-hopped through the DRN nodes to the nearest DDN node. The data is then transmitted by the various DDN nodes to the NCC in parallel. In the simulation, the BSN nodes send their sensed data to their parent DRN node in a periodic manner. The sensing period that was used in the simulation is 10 seconds. This value may be varied depending on the requirements of the application used and the type of parameters sensed. After collecting the information from the BSN nodes, the DRN nodes use the networking protocol to route this information to their parent DRN node. The DRN data packet size was set to 512 bytes, which is a common size and it is sufficient to include the sensed information. For the SRJ algorithm the MAX_JUMP_FACTOR is set to 3. We believe that this is a reasonable value to overcome 3 consecutive node failures by increasing the DRN node transmission range accordingly. To verify and test our RA, and SRJ routing protocols and their ability to route the generated packets correctly to the DDN nodes using intermediate DRN nodes, a number of DRN failures have been intentionally generated using the Poisson arrival distribution with certain average arrival rate.

The average arrival rate of the DRN failures varied from 0 to 4.5 percent of the total number of nodes per month. For example, if the number of DDN nodes is 10, and the number of DRNs per DDN nodes is 100, then the total number of DRN nodes is 1,000. At an average failure rate of three percent per month, the actual number of additional failed nodes for each month is 3% of 1,000 which is 30 nodes. The simulation is designed to evaluate the capability of the routing protocol to overcome intermediate DDN node failures. The failure rate range that is used in these experiments is only theoretical, and is intended to compare the ability of the introduced linear routing protocols to overcome possible node failures. The actual failure rate would be dependent on the application, geographic location, environmental conditions, and specific type and reliability of the devices that are used as DRN nodes as well as the expected device lifetime in the particular environment where the nodes are deployed. The failures may be due to external factors imposed by the physical environmental elements or by internal factors such as hardware or software malfunction due to aging or manufacturing defects. The total simulated time was 20 months.

**Figure 11.**
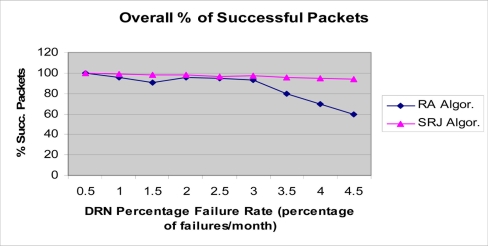
Simulation results for the hierarchical acoustic wireless sensor network. Percentage of successfully transmitted packets. NUM_DRN_PER_DDN = 25.

**Figure 12.**
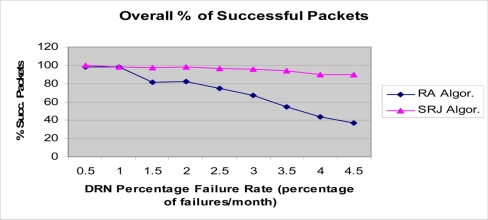
Simulation results for the hierarchical acoustic wireless sensor network. Percentage of successfully transmitted packets. NUM_DRN_PER_DDN = 50.

**Figure 13.**
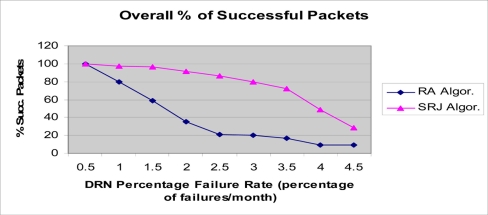
Simulation results for the hierarchical acoustic wireless sensor network. Percentage of successfully transmitted packets. NUM_DRN_PER_DDN = 100.

**Figure 14.**
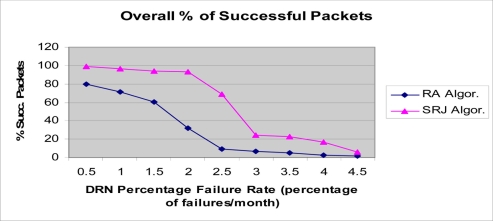
Simulation results for the hierarchical acoustic wireless sensor network. Percentage of successfully transmitted packets. NUM_DRN_PER_DDN = 150.

The results for underwater acoustic wireless sensor networks are presented in the Figures 11–14. As DRNs fail, routing of the DRN packets to either the parent DDN or the alternative one in the opposite direction is done. In Figures 11–14, the number of DRNs per DDN was varied in order to study the impact of increasing the number of DRNs per DDN on network performance. The percentage of successfully transmitted packets was measured as the DRN percentage failure rate (percentage of DRN failures per month) was varied. As can be seen in all four figures the percentage of successfully transmitted packets decreases as the percentage of DRN failures increases. This is consistent with the expected and logical behavior of the system and can further be used to validate the simulation. Also, it can be clearly seen that the SRJ algorithm provides the better performance compared to the RA algorithm respectively. This is expected since the RA algorithm does not try to jump over a failed DRN, and only tries redirecting the packet once. If it encounters another failed DRN in the opposite direction then the packet is dropped. The SRJ algorithm offers a better performance since it considers both directions and dispatches the packet only in the direction with the smallest required energy. In addition to providing more alternatives for overcoming failed DRNs, the SRJ algorithm also ensures a smaller number of DRN failures due to battery depletion which increases network lifetime and improves its performance. Additionally, the results show that as the number of DRNs per DDN increases from 25, to 50, to 150, the percentage of successfully transmitted packets decreases for both algorithms. For example, for the SRJ algorithm case, with a percentage failure rate of 4 percent failures per month, the percentage of successfully transmitted packets decreases from 94.84 for DRN_PER_DDN = 25, to 90.18 for DRN_PER_DDN = 50, to 48.26 for DRN_PER_DDN = 100, to 16.82 for DRN_PER_DDN = 150. This decrease in performance as the number of DRNs per DDN increases is expected due to the linear structure of the network.

With the increased number of DRNs that a packet has to use to reach the DDN, the probability of encountering a more than maximum number of consecutive failed DRNs, which prevents it from going further increases. When designing such a network, the number of DRNs per DDN must not be too large in order to ensure good network performance. Consequently, the choice of the number of DRN per DDN nodes is an important parameter which is affected by the communication range of the protocol used, as well as the desired quality of service (QoS) such as end-to-end delay, bandwidth, and throughput. For example, real-time, audio/video monitoring would have more stringent QoS requirements. This also depends on the application, the particular type of pipeline and the desired monitoring specifications. This is the subject of future research which we plan to investigate in the future.

**Figure 15.**
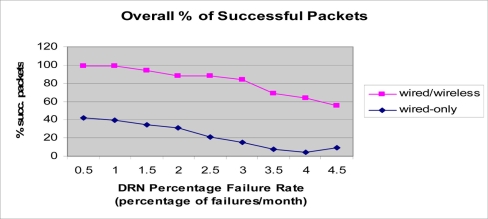
Simulation results for the integrated wired/RF wireless sensor network. Percentage of successfully transmitted packets. NUM_DRN_PER_DDN = 75.

Another simulation experiment was performed for the integrated wired/RF wireless architecture discussed earlier. In the simulation, the same parameters presented in Table 1 were used, except for the fact that a data rate of 1 Gbps was assumed for the wired transmission (fiber optic medium), and 2 Mbps for the wireless transmission. The simulation results presented in Figure 15, clearly demonstrate the increased reliability of the wired/wireless. In this simulation as well, it can be seen that as the failure rate of DRN nodes increases from 0.5 to 4.5 percent, the percentage of successfully transmitted packets decreases from 98.85 to 55.01 percent for the wired/wireless case, and from 42.36 percent to 9.33 percent in the wired-only case. First, the decrease of the percentage of successfully transmitted packets as the failure rate increases is reasonable and provides validation for the simulation. Additionally, the enhanced reliability and superior performance of the wired/wireless architecture is evident in the results. This is due to the fact that a failure in the wired-only architecture prevents messages from being forwarded past the failure point. However, the wired/wireless architecture allows nodes to overcome failures in the wired medium by using the alternative wireless link as a backup which ensures continued propagation of messages even though the propagation on that link is done at a lower rate.

The data rates differ for different architectures. If we assume 1 Gbps for the wired transmission (fiber optic medium), 2 Mbps for the RF wireless transmission, and 4,800 bps for underwater acoustic wireless transmission are used. Then the data rate is 1 Gbps for underwater wired sensor network without faults and it drops to 0 if there is a fault. However, if we use integrated wired/acoustic wireless sensor network then the data rate is normally 1 Gbps and with a fault this will drop to only a maximum of 4,800 bps by relying on the acoustic nodes to relay the messages past the failure points. Similarly, if the integrated wired/RF wireless sensor network is used then the data rate will be 1 Gbps without faults and 2 Mbps with the faults.

## 7. Discussion

The sensor network architectures for monitoring underwater pipeline mentioned in this paper provide different advantages for different objectives and requirements. Wired sensor networks usually provide higher communication bandwidth than wireless sensor networks and underwater acoustic wireless sensor networks. While optical networks for wide areas can provide high bandwidths such as 4,476 Mbps for OC-96, 9,953 Mbps for 10-Gigabit Ethernet WAN PHY, 13,271 Mbps for OC-256, and 39,813 Mbps for OC-768/STM-256, wireless communication networks cannot reach these figures [37]. For example, the bandwidth is 0.250 Mbps for Zigbee, 24 Mbps for Bluetooth 3.0, 70 Mbps for WiMax, and 600 Mbps for IEEE 802.11n. This is due to the existence of noise and the high cost of signal collusions. The bandwidths are farther reduced significantly with acoustic communication in underwater networks. Using both high bandwidth interconnection devices and high bandwidth wired links, the network can be used for pipeline monitoring applications that demand high bandwidth such as camera-based monitoring. On the other hand, wireless/acoustic wireless networks provide better reliability for pipeline monitoring applications such as sudden damages detection. These applications are event based applications that do not need high communication bandwidth. Events occur only if there are exceptions discovered by the sensors which they need to communicate. The integrated wired/RF wireless sensor network and integrated wired/acoustic wireless sensor network architectures can provide reasonable bandwidth as well as good reliability for event-based applications even if some parts of the network are damaged.

The network architectures mentioned in this paper also have different degrees of reliability. The wired network may fail with a single point failure. On the other end, using wide transmission ranges in RF wireless and underwater acoustic wireless networks can solve the problem of a network failure due to failure of one or more nodes. The reliability degree of the wireless network can be increased as we increase the transmission range. However, this increase of the wireless transmission range causes a significant increase in power consumption. Therefore, the battery will be consumed rapidly and the life of the network will be reduced significantly. In the integrated wired/wireless networks and the integrated wired/acoustic wireless networks, the limitation of power sources is not an issue. Therefore, the node can be designed to use a wide transmission range for communication to provide better reliability.

Some network architectures discussed earlier depend on surface buoys. These architectures are RF wireless sensor networks and integrated wired/RF wireless sensor networks. Using buoys connected through wires to an underwater pipeline in very deep sea is not practically feasible since the wires will be vertically expanded for a long distance (from the depth of the pipelines to the surface) which will significantly increase the probability of damages. Considering this important point, using integrated wired/acoustic sensor networks could provide better reliability in deep sea situations.

Further to the benefit of increasing the reliability of the network, the availability of wireless nodes that are embedded in the wired networks can be used for other applications. One possible application is using external RF wireless or underwater acoustic wireless sensor nodes to monitor some aspects of the pipeline such as monitoring if there is any movement, sound, or hazards around the area of pipeline. The external nodes can send their information to the NCC through the integrated wired/RF wireless network or the integrated wired/acoustic wireless network. The integrated wired/wireless network represents the backbone for transferring the collected information to and from the NCC. The external nodes can be inexpensive sensor nodes with limited capabilities and scattered randomly. The external nodes can also be moving vehicles that need to communicate with the pipeline infrastructure. One example of using this type of communication is with maintenance boats or underwater robots that need to collect more information about the pipeline status. Another example is security vehicles that need to send special signals to the network not to report the movement of the vehicle to the NCC in case the pipeline infrastructure is equipped with motion sensors that automatically report approaching vehicles. Although the external nodes option can also be made available with completely wireless sensor networks for monitoring pipeline infrastructures, the existence of a wired network can provide higher communication bandwidth and reliability. A summary of the reliability issues for all discussed architectures is in Table 2.

The hierarchical model presented in this paper employs a heterogeneous architecture with three types of nodes. It is worth noting that a homogeneous model can be used to perform the required monitoring and communication. In such a model, there would be only sensor nodes which are used to provide both sensing and routing services. Even though a self-organizing homogeneous model would have the following advantages: (1) Lower system cost; (2) Reduced installation complexity and time. The three-level heterogeneous model that we present has the following advantages: (1) Increased reliability: in the homogeneous network data messages would have to travel along the entire length of the network in order to reach the sink while in the three-level model, the messages are disseminated to the NCC by the DDN nodes at regular geographic intervals. Therefore, a disruption in the communication in one of the segments between two DDN nodes would not affect the transmission of messages in the other segments. This leads to higher overall system reliability and robustness; (2) Lower end-to-end delay: This is also due to the fact that in the heterogeneous system, the messages have to travel shorter distances before being disseminated to the NCC; (3) Reduced energy consumption (in Joules per byte) by the sensor nodes, which is critical for the important objective of longer network lifetime and maintenance. This is due to the fact that in the homogeneous network, the same data message would have to use many more hops to reach the destination. This requires significantly increased energy consumption at the individual sensors; (4) Increased bandwidth capabilities and improved performance. This is the case because, in the heterogeneous model, DDN nodes transmit data to the NCC in a parallel fashion. Therefore, the communication bandwidth of a particular DDN node is ultimately shared by a lower number of sensor nodes, which are in the segment that is serviced by that node.

Consequently, while the homogeneous model can still be used in cases where the geographic area and length of the pipeline infrastructure is relatively shorter, most underwater pipeline systems stretch for very long distances, where the heterogeneous model would be better suited to meet typical system requirements of improved reliability and performance, reduced end-to-end delay, decreased energy consumption by the sensor nodes, and increased network lifetime.

## 8. Conclusions and Future Work

This paper developed, discussed and evaluated a number of sensor networks architectures for monitoring underwater pipelines. The reliability issues of five sensor network architectures for monitoring long underwater pipelines were discussed. These architectures are underwater wired sensor networks, underwater acoustic wireless sensor networks, RF wireless sensor networks, integrated wired/acoustic wireless sensor networks, and integrated wired/RF wireless sensor networks. The paper developed an analytical model to compare the reliability of different architectures for monitoring underwater pipelines. The newly introduced integrated wired/wireless networks can provide good reliability in terms of network connectivity, continuity of power supply, and physical network security. In this architecture, the node batteries will be used only if there is a fault in the wires providing power to the nodes. Unlike wireless sensor networks, if there is no cut for some period, the batteries will be fully charged during that period. As a result nodes will have better chance to have power supply in integrated wired/wireless sensor network than wireless sensor networks. In addition integrated wired/wireless sensor networks can have less physical security concerns than underwater wired sensor networks as physical attacks on the network will not stop the connectivity of the network in some degree. Although the integrated wired/wireless sensor network can provide good sensing and communication reliability, it is a new technology that may face some challenges in the design and implementation. As a result and as future work, we plan to design, analyze and evaluate in details the integrated wired/wireless sensor network architecture for monitoring long underwater pipelines. We plan to study the feasibility and effectiveness of this network. We intend to study the implementation issues of the mentioned networks as well as evaluating different technologies that can be used to implement these architectures. In addition, we plan to develop and evaluate cross-layered optimizations to enhance communication in the proposed architectures.

## Figures and Tables

**Table 1.** Case study simulation parameters.

**Parameter**	**Value**
Total Number of DDNs	10
Total Number of DRNs Per DDN	25, 50, 100, 150
Total Number of BSNs Per DRN	6
Periodic Sensing Interval	10 seconds
Data transmission rate	4,800 bps
DRN Data Packet Size	512 bytes
MAX JUMP FACTOR	3

**Table 2.** A Summary of the reliability issues for different sensor network architectures for underwater pipelines.

**#**	**Architecture**	**BW**	**BWF**	**EC**	**PSC**
1	Underwater wired sensor network	High	0	No	High
2	Underwater acoustic wireless sensor network	Low	Low	Yes	Low
3	RF Wireless sensor network	Med	Med	Yes	High
4	Integrated wired/acoustic wireless sensor network	High	Low	No	Low
5	Integrated wired/RF wireless sensor network	High	Med	No	Low

BW: Bandwidth; BWF: Bandwidth with fault condition; EC: Energy constrains; PSC: Physical security concern.
